# Forced Migration and Global Responsibility for Health: Comment on "Defining and Acting on Global Health: The Case of Japan and the Refugee Crisis"

**DOI:** 10.15171/ijhpm.2016.146

**Published:** 2016-11-05

**Authors:** Kayvan Bozorgmehr, Oliver Razum

**Affiliations:** ^1^Department of General Practice and Health Services Research, University Hospital Heidelberg, Heidelberg, Germany.; ^2^Department of Epidemiology and International Public Health, School of Public Health, Bielefeld University, Bielefeld, Germany.

**Keywords:** Forced Migration, Social Protection, Right to Health, Global Health, Humanitarian, Internally Displaced, Border Control, Securitization, Solidarity

## Abstract

Forced migration has become a world-wide phenomenon in the past century, affecting increasing numbers of countries and people. It entails important challenges from a global health perspective. Leppold et al have critically discussed the Japanese interpretation of global responsibility for health in the context of forced migration. This commentary complements their analysis by outlining three priority areas of global health responsibility for European Union (EU) countries. We highlight important stages of the migration phases related to forced migration and propose three arguments. First, the chronic neglect of the large number of internally displaced persons (IDPs) in the discourses on the "refugee crisis" needs to be corrected in order to develop sustainable solutions with a framework of the Sustainable Development Goals (SDGs). Second, protection gaps in the global system of protection need to be effectively closed to resolve conflicts with border management and normative global health frameworks. Third, effective policies need to be developed and implemented to meet the health and humanitarian needs of forced migrants; at the same time, the solidarity crisis within the EU needs to be overcome. These stakes are high. EU countries, being committed to global health, should urgently address these areas.

Forced migration refers to a migratory movement (between or within a country) in which an element of coercion exists, including threats to life and livelihood arising from natural or man-made causes.^1^ This form of migration has become a phenomenon affecting millions of people since the 1940s, and an increasing number of countries in the past decades. Nine in every 1000 inhabitants of the world were forcibly displaced in 2015.^2^ The recent peak in forced displacement has been labelled as a “refugee crisis,” a term defining the victims as the problem, instead of problematizing the underlying causes of displacement. The true crisis is, as United Nations (UN) Secretary General Ban Ki Moon has put it, rather a crisis of solidarity.^2^ It is also not an acute but a chronic crisis: the number of displaced persons has already been at very high levels around 6 to 7 per 1000 world population between 1996 and 2013.^2^ Despite its long-lasting character and its obvious implications for population health, forced migration has not been explicitly categorised as a global health issue.^3^ On the contrary: responses to the health and humanitarian needs arising from the high number of forcibly displaced persons have been deeply unsatisfactory. The international community not only fails to develop a coherent multilateral strategy to enhance protection^4,5^; moreover, many nation states respond with restrictions^6^ instead of serving the health and humanitarian needs of refugees and asylum-seekers.

As Leppold et al note in their perspective on Japan’s role in global health, the “refugee crisis” disguises a blatant mismatch between government action and the rhetoric on global responsibility in health that accompanies global health discourses.^3^ Many countries, such as Switzerland, the United Kingdom, Norway, Japan, Sweden, and Germany, as well as the European Union (EU), have affirmed their commitment to global health in conceptual strategy papers.^7^ Although global health entails different meanings,^8^ all strategies share the rhetoric of a rights-based approach to health and the global responsibility for health.

What does global responsibility for health mean in the context of forced migration? We here complement the Japanese perspective provided by Leppold et al with the perspective we believe the EU should take. For this purpose, we highlight important stages of the migration phases related to forced migration and then outline three priority areas of responsibility for health for EU countries.

## Internal Displacement and Sustainable Solutions

The discourse on the “refugee crisis” in many recipient or transit countries of the EU focuses only on refugees or asylum-seekers reaching *the EU*.^9^ However, of those persons forcibly displaced in 2015 (65.3 million according to United Nations High Commissioner for Refugees, UNHCR^2^), the majority (62.5%) have been internally displaced persons (IDPs). In 2014, 77% of the world’s IDPs (38 million) lived in just 10 countries: Syria (19.9%), Colombia (15.8%), Iraq (8.6%), Sudan (8.1%), DR Congo (7.2%), Pakistan (5,0%), South Sudan (3.9%), as well as Somalia, Nigeria, and Turkey (each < 3.0%). The political and policy discourses in the EU on health and humanitarian needs of forcibly displaced people, thus, only affect a fraction of those in need.

Since the current humanitarian system is not adequately set up to support IDPs, host communities increasingly bear the task of assisting them.^10^ These are mostly fragile states with limited coping capacity, in which large numbers of IDPs put additional strain on scarce resources, increasing the risk for further conflict and displacement.^10^ Chronicity is a recurring theme in this context: in 90% of the 60 countries monitored by the Internal Displacement Monitoring Centre, people were living in displacement for 10 years or more.^10^ Excess mortality in complex emergencies appears to be highest in IDPs compared to residents or migrants with refugee status.^11^ IDPs are hard to reach by humanitarian organisations and not protected by a specific status.^10^ They hence face substantial barriers to meet existential needs such as access to food, water, shelter, and health services.^11,12^

The Sustainable Development Goals (SDGs) reflect the human development agenda of the international community until 2030. Many of the SDGs, especially SDGs 1-8, are highly relevant from a rights-based approach to sustainable solutions^13^ for the high number of IDPs worldwide. The SDGs do not, however, take account of the phenomenon of forced migration. Migration appears explicitly only in SDG 10 with a reference to implementing planned and well-managed migration policies and reducing costs of sending remittances.

EU states should - and could - do more than that. They should apply the generic SDGs within a human rights framework to the situation of IDPs. This could become the key for EU-led global health and humanitarian assistance programs to improve the situation of IDPs and take on responsibility for health.

## Safe Passage, Securitisation and Human Rights

For more than a decade, the EU has been working towards an integrated border management,^14^ which primarily consists of policing, securitisation,^15,16^ and more recently also off-shoring and out-sourcing,^17^ of activities aimed at fending off immigrants. This practice has negative implications for migrants in transit^18^ whose health and humanitarian needs are not adequately met, and often enough actively violated.^19^ The existing legal systems of protection, including the Geneva Convention on the Status of Refugees and the European Convention for the Protection of Human Rights and Fundamental Freedoms, are poorly adapted to the present realities of migration flows.^20^ It suffers from two main shortcomings: (*i*) “protection gaps,” reflecting unmet needs of forced migrants falling outside the conventional status of asylum-seekers or refugees; and (*ii*) a limited “protection space” with respect to geographical locations in which migrants in need are found,^21^ such as the sea. One of the most blatant examples of these shortcomings is the EU response to needs of migrants crossing the Mediterranean,^5^ which – according to the German Institute of Human Rights, Berlin, Germany – have been in conflict with human rights in several dimensions already well before 2015.^22^ They are not provided safe passage, which indicates that a global social policy which considers health and humanitarian needs is not enforced.^23^ Currently, EU border management and the implementation of supra-territorial social policies and human rights conflict with each other.^24^ Also, the mantra of universal health coverage, inevitably linked to health as human right, is incompatible with the securitizing^24^ and politicising^25^ response of the EU. Member states, being committed to global health, urgently need to resolve these conflicts and close normative, legal and policy gaps^21^ to meet their responsibility for health.

## Meeting Social Needs and Overcoming European Union’s Solidarity Crisis

Forced migrants reaching destination countries in the EU face an absence of a harmonized approach to existential needs, such as food, housing, healthcare,^14^ or access to the labour market. Countries restricting entitlements^6^ or those providing sub-standard services increase the pressure on forced migrants to seek a better future in other countries which provide higher standards or uphold human rights. The lack of solidarity leads to a race to the bottom with respect to entitlements to services for existential needs. To increase solidarity, the EU promotes and tries to implement an EU-wide relocation to increase ‘fairness’ in tackling the perceived economic and societal burden related to accepting forced migrants. This policy instrument is not only a technocratic approach to “solidarity” which conflicts with the freedom of movement.^26^ According to media reports it is also failing in practice: of the 160 000 refugees and asylum-seekers who were to be relocated in 2015, less than 5000 have been actually transferred within the EU within one year.^27^ Some of them migrated back to the particular EU country which they were supposed to leave,^27^ suggesting that migrants have agency and migration cannot be stopped by technocratic approaches.^26,28^

What can a policy look like that increases solidarity within the EU, respects freedom of movement, and avoids a race to the bottom with respect to entitlements to services for existential needs? Elements of an appropriate policy instrument can be found in the idea of a global fund for health which – in a nutshell – consists of revenue collection and pooling of funds for health and healthcare beyond territories.^29,30^ A similar approach to funding and financing services for existential needs of forced migrants could meet the above criteria: funds would be raised across the EU according to an ability-to-pay criterion, financial flows would be directed towards member states based on the number and the social needs of forced migrants they host. Linked to an EU-wide mechanism for asylum application, a *post hoc* mechanisms that financially recompenses receiving countries based on the social and health needs of the forced migrants they host would make forced relocations and restrictions on the freedom of movement dispensable.^26^ This requires not only political commitment, but also the power of law^31^ to enforce responsibility for health across territories. Obviously, this system would need to be expanded towards a global social protection mechanism in order to reduce structural and external pressure to migrate – thus, offering Japan additional means of international engagement for refugees beyond those described by Leppold et al.

## Conclusions

As we have shown, global responsibility for health in the context of forced migration entails substantial challenges. EU countries committed to global health need to address three priority areas: (*i*) effectively and sustainably improve the situation of IDPs and correct the chronic neglect of IDPs in discourses on forced migration; (*ii*) close the gaps in the global protection system as well as resolve the unacceptable conflict between border management and global health aspirations (such as health as human right and universal health coverage); and (*iii*) develop and implement effective social policies to overcome the solidarity crisis within the EU in meeting health and humanitarian needs of forced migrants. These would comprise international mechanisms for funding and financing services to existential needs of forced migrants based on a solidarity principle, and channelling resources according to the movement of refugees.

## Ethical issues

Not applicable.

## Competing interests

Authors declare that they have no competing interests.

## Authors’ contributions

KB: conceived the commentary, wrote the first draft. OR: revised the manuscript for important intellectual content. Both authors read and approved the final version for publication.

## Authors’ affiliations

^1^Department of General Practice and Health Services Research, University Hospital Heidelberg, Heidelberg, Germany. ^2^Department of Epidemiology and International Public Health, School of Public Health, Bielefeld University, Bielefeld, Germany.
